# Serological Evidence of Hantavirus Infection in Apparently Healthy People from Rural and Slum Communities in Southern Chile

**DOI:** 10.3390/v7042006

**Published:** 2015-04-17

**Authors:** Claudia Muñoz-Zanzi, Farides Saavedra, Carola Otth, Ljubica Domancich, Melissa Hott, Paula Padula

**Affiliations:** 1 Division of Epidemiology and Community Health, School of Public Health, University of Minnesota, 1300 S. Second Street, suite 300, Minneapolis, MN 55454, USA; 2 Institute of Animal Pathology, Faculty of Veterinary Medicine, Austral University of Chile, Campus Isla Teja, P.O. Box 567, Valdivia, Chile; 3 Institute of Clinical Microbiology, Austral University of Chile, Campus Isla Teja, P.O. Box 567, Valdivia, Chile; E-Mails: farides.saavedra@gmail.com (F.S.); cotth@uach.cl (C.O.); ljubica.domancich@gmail.com (L.D.); melissahottogalde@hotmail.com (M.H.); 4 National Institute of Infectious Diseases, ANLIS Dr. Carlos G. Malbran, Velez Sarsfield 563 CABA (1281), Buenos Aires 1281, Argentina; E-Mail: ppadula@gmail.com

**Keywords:** hantavirus, ANDV, sero-prevalence, peri-domestic rodents, Chile, ELISA

## Abstract

Hantavirus disease in America has been recognizable because of its rapid progression in clinical cases, occurrence in previously healthy young adults, and high case fatality rate. Hantavirus disease has been proposed now to define the diversity of clinical manifestations. Since 1995, a total of 902 cases of hantavirus pulmonary syndrome have been reported in Chile, caused by Andes virus (ANDV), with overall fatality of 32%. This report describes the sero-epidemiology of hantavirus in apparently healthy people in rural and urban slum communities from southern Chile. Ten of 934 samples yielded a positive result resulting in a seroprevalence of 1.07% (95% confidence intervals: 0.05%–2.0%). A higher proportion of positive samples was found among individuals from rural villages (1.3%) and slums (1.5%) compared with farms (0.5%). Seropositivity was associated with age (*p* = 0.011), low education level (*p* = 0.006) and occupations linked to the household (homemaker, retired, or student) (*p* = 0.016). No evidence of infection was found in 38 sigmodontinae rodents trapped in the peri-domestic environment. Our findings highlight that exposure risk was associated with less documented risk factors, such as women in slum and rural villages, and the occurrence of infection that may have presented as flu-like illness that did not require medical attention or was misdiagnosed.

## 1. Introduction

The *Hantavirus* genus, *Bunyaviridae* family, includes several zoonotic viruses that are maintained in rodent reservoirs [1]. Humans are incidental hosts and are typically infected via contaminated aerosolized secretions (feces, urine, saliva) of the reservoir animals [2]. The first report of human hantavirus infection in the Americas documented serologically-confirmed cases that occurred in Brazil in 1990 [3]. Hantavirus cardiopulmonary syndrome (HCPS) often cause considerable alarm because of the rapid disease progression in some clinical cases, its occurrence in previously healthy young adults, and the high case fatality rate [4]. Recently, the more inclusive name Hantavirus disease has been proposed to define the clinical manifestation of infection instead of the traditionally used hemorrhagic fever with renal syndrome (HFRS) and hantavirus cardiopulmonary syndrome (HCPS) due to better understanding of the diversity of clinically overlapping manifestations beyond two distinct syndromes and the occurrence of mild or asymptomatic infection that may go underdiagnosed [5,6]. 

Hantavirus infection was first recognized in Chile in 1995 with Andes virus (ANDV) South as the primary lineage identified to date [7,8]. The ANDV clade is comprised of at least 12 variants found in six countries (Argentina, Bolivia, Brazil, Chile, Paraguay, Peru, and Uruguay) and harbored by Akodon, Necromys and Oligoryzomys rodents [9]. Human-to-human transmission has been reported only in Argentina and Chile [10]. Since 1995, a total of 902 cases of hantavirus pulmonary syndrome (HPS) have been diagnosed in Chile. Surveillance records reported 54 cases of HPS and three reported cases of mild presentation in 2014, with overall fatality of 31.5% [11]. This report describes the sero-epidemiology of hantavirus in apparently healthy people in distinct community types from Southern Chile.

## 2. Material and Methods 

Individuals from 12 communities in the Los Rios Region of Chile (Latitude: 39°15’S–40°33’S, Longitude: 73°43’W–71°35’W) were invited to participate in a series of studies on the eco-epidemiology of zoonotic infections. Communities, two of each community type, were selected based on the following definitions: (i) Slums (U): informal settlements near an urban area characterized by high density of substandard housing, (ii) Villages (C): rural community settlements away from major cities where households are clustered together, (iii) Farms (D): dispersed households, typically small family farms, located in a specific rural locality. Details of the study area and protocols have been previously described [12,13]. Up to 40 representative households from each community were enrolled and all household members of at least 13 years of age were invited to participate. Information collected included basic demographic characteristics from individual participants and living conditions at the household level. As part of the larger study, rodents were trapped over three nights from within the house and the peri-domestic area. The study protocol was approved by the University of Minnesota’s IRB (No. 0903M62042) and IACUC (No. 0904A63201) and the Austral University’s Human and Animal Ethics Committee (No. 01/09). Frozen serum samples from 934 people were tested for presence of ANDV hantavirus IgG antibodies at dilutions of 1:400 using a previously published method [14]. A total of 393 rodents were trapped in the same study communities as part of a previous study. Of those, 38 banked frozen serum samples from sigmodontinae species were available for testing (20 *Oligoryzomys longicaudatus*, 4 *Akodon longipilis*, and 14 *Abrothix* spp.) [12]. For rodent samples, because of the low volume, only a preliminary testing was carried out for presence of ANDV IgG antibodies at dilution of 1:200 [15]. Positive samples were classified based on a cutoff of ≥0.3 for the difference in the absorbance between the ANDV antigen and non-specific antigen [14]. Samples positive at first screening were centrifuged and re-tested at dilutions of 1:400 and 1:800 for human samples and of 1:200 and 1:400 for rodent samples. Samples that tested positive at re-testing were considered positive. All laboratory tests were carried out at the local Hantavirus Reference Laboratory, Austral University of Chile. An initial evaluation of the random effects by household and community did not justify the use of mixed-effects logistic regression (lme4 package) for analysis. Consequently, and for simplicity, as multivariable analysis was not feasible due to small sample, the statistical significance of the association between various demographic and living conditions factors and the serologic status of study participants was examined using Fisher exact methods. Statistical significant was set at *p* < 0.05. Ninety-five percent confidence intervals (c.i.) for the sero-prevalence estimates were obtained using exact methods. All statistical analyses were carried out using the R 2.15.1 statistical program. 

## 3. Results and Discussion 

Ten of the 934 samples yielded a positive result resulting in a sero-prevalence of 1.07% (95% c.i.: 0.05%–2.0%). This overall prevalence among the apparently healthy study population was higher than the sero-prevalence of 0.27% obtained from a national sero-prevalence study of IgG antibodies to ANDV in 2891 people using a strip immunoassay technique [16]. The estimate is similar to an estimate of 1% in Southern Argentina [17], but lower than the estimates of 2.3% to 3.5% in Southern Brazil [18] and 1.7% in Peru [19] and Venezuela [20]. Much higher sero-prevalence estimates has been reported in other regions from South America including 9.1% [21] and 12.2% [22] in Bolivia and 13.5% in Colombia [23]. Estimates are not directly comparable as they are likely influenced by the representativeness of the study populations, laboratory methods, and the distribution of characteristics associated with exposure. Table 1 shows the distribution of study population characteristics and the description of positivity by each level of the variables examined. The 10 sero-positive samples were distributed across eight communities with two communities, a slum community (U-2) and a farm community (D-3), yielding two positive individuals each. Only one of the four farm communities had positive results (Table 2). Proportion of positive samples increased with age with most of the positive samples coming from participants aged ≥66 years (*p* = 0.011). This association between IgG seropositivity and older age is probably a result of population cumulative exposure and is consistent with the national survey that reported an increasing sero-prevalence from 0% in the 17–24 years-old stratum to 1.53% in the ≥65 years-old stratum [16]. The youngest positive individual corresponded to a 13 year-old boy from a slum community. Sero-positivity was significantly associated with having an education up to 4^th^ grade only (*p* = 0.006) and, although not statistically significant (*p* = 0.444), a higher proportion of positive samples was found among individuals from rural villages (1.3%) and slums (1.5%) compared with farms (0.5%). Considering the small number of seropositive individuals, we were not able to adjust the analysis for potential confounders; however, older people also had lower education levels (94% of the people in the ≥66 years old category completed only up to 8^th^ grade) and were more likely to be residents of rural villages (49% of the people in the ≥66 years old category lived in rural villages) which could explain the observed associations. Six of the 10 positive samples corresponded to women who reported “homemaker” as occupation and sero-positivity was significantly associated with people reporting occupations linked to the household (homemaker, retired, or student) (*p* = 0.016). Exposure to a hantavirus-contaminated environment during work on farms, specific outdoor occupations, and outdoor recreational activities are commonly documented risk factors for infection [24], yet, nine of the 10 seropositive individuals were children, housewives or elderly who spent more time in the household environment. Additionally, there was higher evidence of prior infection in slum and village communities (~1.5%) compared with farms (0.5%). The opposite has been documented in Chile [25,26] but it is consistent with other reports such as a Peruvian amazon study contrasting an urban population (2.2%) and rural (1.1%) population [19]. Because the observed IgG seropositivity reflects evidence of exposure sometime in the past, we cannot pinpoint to specific infection sources; however, the greater risk associated with the domestic environment could be due to routine activities (*i.e.*, clearing weeds, cleaning outbuildings, use of food storage areas, *etc*.) carried out in areas where sigmodontine rodents approach the peri-domestic environment in search for food. Although testing of the 38 wild rodents did not yield positive results, wild species potentially carriers of hantavirus, were trapped in each community type, including slums. The slum communities in the study region are relatively small, from 45 to 265 households, and surrounded by natural vegetation which likely facilitates this wild-domestic habitat contact. This study did not test human or rat samples for evidence of SEOV infection; however, considering the presence of murine rodents in close proximity to the households, the higher sero-prevalence in women and other people linked to the domestic environment, and the potential for assay cross-reactivity between different hantavirus serotypes [27,28], it is likely that the study communities are exposed to murine-SEOV as well. Furthermore, there was no self-reported history of hantavirus diagnosis among the study population and occurrence of infection may have presented as flu-like illness that did not require medical attention or was misdiagnosed [29]. Flu-like illness is more compatible with mild SEOV infections than HCPS infections [5]. These findings highlight the need to expand diagnostic criteria and surveillance programs to improve our understanding of the ecology of circulating hantavirus strains beyond ANDV in the region and their associated public health burden. 

**Table 1:** Description of study population and group-specific seroprevalences of hantavirus in Los Rios, Chile 2010-2012.

Variable		Distribution of study participants by level of variable (n = 934)	No. sero-positive (Prevalence by level of variable)	*p*-value
Community type	Rural village	302 (32.3%)	4 (1.3%)	0.444
	Farms	365 (39.1%)	2 (0.5%)	
	Urban slums	267 (28.6%)	4 (1.5%)	
Sex	Female	534 (57.2%)	6 (1.1%)	1.0
	Male	400 (42.8%)	4 (1.0%)	
Age (years)	13 to 18	100 (10.7%)	1 (1.0%)	0.011
	19 to 35	295 (31.6%)	1 (0.3%)	
	36 to 65	423 (45.3%)	3 (0.7%)	
	≥ 66	116 (12.4%)	5 (4.3%)	
Occupationⱡ	Construction work	40 (4.3%)	1 (2.5%)	0.051
	Domestic/students	549 (59.7%)	9 (1.6%)	
	Other occupation *	331 (36.0%)	0	
Education	Up to 4^th^ grade	156 (17.0%)	6 (3.8%)	0.006
	5^th^ to 12^th^ grade	700 (76.4%)	4 (0.6%)	
	Technical/college	60 (6.6%)	0	
Incomeⱡ	Very low	568 (60.8%)	8 (1.4%)	0.331
	Low	366 (39.2%)	2 (0.5%)	
Gardening	Yes	283 (30.3%)	4 (1.4%)	0.500
	No	651 (69.7%)	6 (0.9%)	
Cleaning barns	Yes	344 (36.8%)	0	0.017
	No	590 (63.2%)	10 (1.7%)	

* Other occupations included agricultural work, other outdoor work, and office work; ⱡ Missing data: 12 people for Occupation and 18 people for Income.

**Table 2:** ELISA Optical Density (OD) results of a total of 934 participants from 12 communities who tested positive for ANDV IgG antibodies. Samples with OD ≥ 0.3 are considered positive.

ID	Community ID *	Sex	Age	Occupation	Education level	OD 1:400
1	U-1	M	13	Domestic	5th to 12^th^	1.142
2	U-2	F	71	Domestic	≤4^th^	3.564
3	U-4	F	43	Domestic	5^th^ to 12^th^	0.820
4	U-4	F	33	Domestic	5^th^ to 12^th^	1.189
5	C-1	M	86	Domestic	≤4^th^	0.689
6	C-2	F	39	Domestic	≤4^th^	1.945
7	C-3	F	74	Domestic	≤4^th^	0.368
8	C-4	M	49	Construction	5^th^ to 12^th^	0.889
9	D-3	F	75	Domestic	≤4^th^	0.521
10	D-3	M	79	Domestic	≤4^th^	3.117

* C: Rural village; D: farm; U: urban slum.
